# Unusual Presentation of a Sigmoid Mass with Chicken Bone Impaction in the Setting of Metastatic Lung Cancer

**DOI:** 10.1155/2019/1016534

**Published:** 2019-06-26

**Authors:** Ziad Zeidan, Zarnie Lwin, Harish Iswariah, Sheyna Manawwar, Anitha Karunairajah, Manju Dashini Chandrasegaram

**Affiliations:** ^1^School of Medicine, University of Exeter, Exeter, Devon, UK; ^2^School of Medicine, The University of Queensland, Brisbane, Queensland, Australia; ^3^Department of Medical Oncology, The Prince Charles Hospital, Brisbane, Queensland, Australia; ^4^Department of General Surgery, The Prince Charles Hospital, Brisbane, Queensland, Australia; ^5^Department of Pathology, The Prince Charles Hospital, Brisbane, Queensland, Australia

## Abstract

**Background:**

Ingestion of foreign bodies can cause various gastrointestinal tract complications including abscess formation, bowel obstruction, fistulae, haemorrhage, and perforation. While these foreign body-related complications can occur in normal bowel, diseased bowel from inflammation, strictures, or malignancy can cause diagnostic difficulties. Endoscopy is useful in visualising the bowel from within, providing views of the mucosa and malignancies arising from here, but its ability in diagnosing extramural malignancies arising beyond or external to the mucosa of the bowel as in the case of metastatic extramural disease can be limited.

**Case Summary:**

We present the case of a 60-year-old female with an impacted chicken bone in the sigmoid colon with formation of a sigmoid mass, on a background of metastatic lung cancer. On initial diagnosis of her lung cancer, there was mild Positron Emission Tomography (PET) avidity in the sigmoid colon which had been evaluated earlier in the year with a colonoscopy with findings of diverticular disease. Subsequent computed tomography (CT) scans demonstrated thickening of the sigmoid colon with a structure consistent with a foreign body distal to this colonic thickening. A repeat PET scan revealed an intensely fluorodeoxyglucose (FDG) avid mass in the sigmoid colon which was thought to be inflammatory. She was admitted for a flexible sigmoidoscopy and removal of the foreign body which was an impacted chicken bone. She had a fall and suffered a fractured hip. During her admission for her hip fracture, she had an exacerbation of her abdominal pain. She developed a large bowel obstruction, requiring laparotomy and Hartmann's procedure to resect the sigmoid mass. Histopathology confirmed metastatic lung cancer to the sigmoid colon.

**Conclusion:**

This unusual presentation highlights the challenges of diagnosing ingested foreign bodies in patients with metastatic disease.

## 1. Introduction

Around 20% of ingested foreign bodies fail to pass through the gastrointestinal tract [1]. These can result in complications such as abscess formation, bowel obstruction, fistulae, haemorrhage, and perforation [2]. These complications can present in a variety of different clinical scenarios. The purpose of this case report was to highlight a scenario in which an ingested foreign body may present, and to outline the challenges of reaching the diagnosis, along with outlining the possible limitations of endoscopic investigations in diagnosing a colonic malignancy.

Our patient had an impacted chicken bone in the sigmoid colon in the setting of metastatic non-small-cell lung cancer. This was investigated radiologically and found to be an intensely FDG-PET avid mass, initially presumed to be either an inflammatory mass related to the chicken bone impaction or metastatic disease related to her lung cancer. This mass appeared to resolve upon removal of the chicken bone; however, she represented later with a subacute large bowel obstruction related to the sigmoid mass which was found to be metastatic lung cancer at surgery. Consequently, our case highlights the difficulties of establishing a diagnosis in this complex case.

In this case report, we present a literature review of colonic chicken bones and investigate similar patterns across the various presentations reported. PubMed and Google Scholar were both utilised to identify the search terms “chicken bone” AND “bowel” OR “large bowel” OR “colon”. The results were systematically reviewed to include only case reports of chicken bones in the large bowel, while the details of each case were analysed for the purposes of the literature review.

## 2. Case Presentation

We present the case of a 60-year-old lady who initially presented with a pseudomonas empyema and a right hilar mass. Initial diagnostic bronchoscopy revealed no endobronchial lesion. She was treated under the respiratory and infectious diseases' teams with decortication and antibiotics which resulted in marked clinical improvement. Follow-up imaging showed a persistent right hilar mass, necessitating a repeat diagnostic bronchoscopy and biopsy. This revealed a non-small-cell lung cancer (NSCLC) adenocarcinoma which was EGFR and ALK negative.

**Figure 1:**
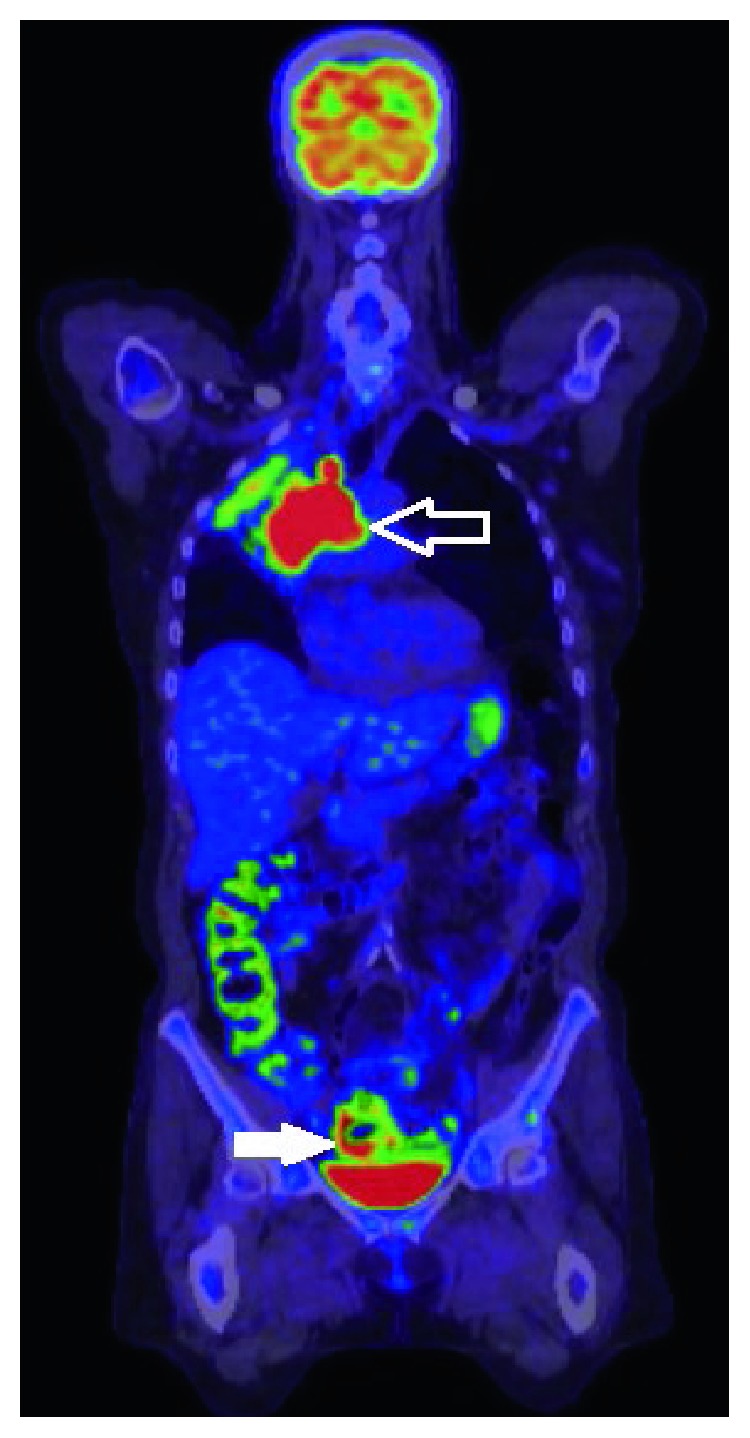
FDG-PET scan with an extensive right upper lobar and mediastinal mass in keeping with primary non-small-cell lung cancer (arrow). Intense heterogenous uptake in the sigmoid colon (white arrow), which could represent a synchronous malignancy or complication secondary to diverticular disease.

**Figure 2:**
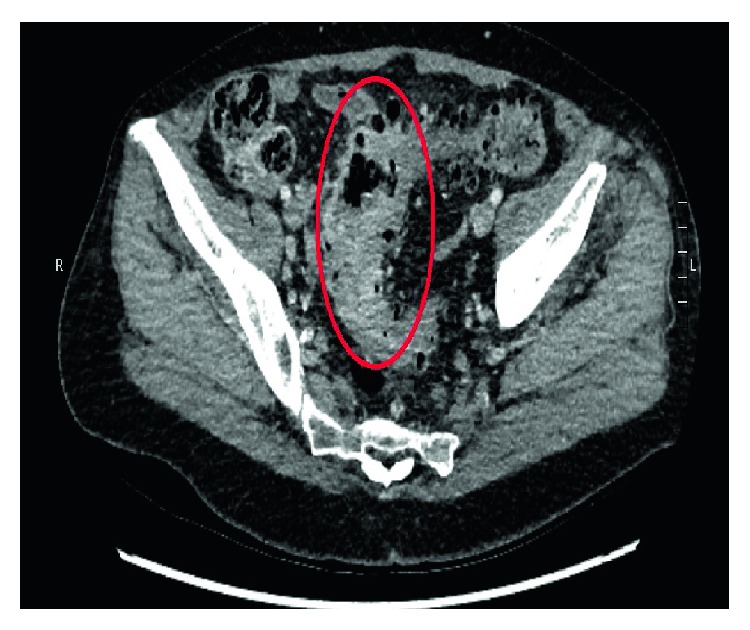
Axial CT highlighting a focal area of thickening in the wall of the sigmoid colon with surrounding diverticula.

Baseline staging imaging revealed that she had metastatic disease with a right lung primary lesion, mediastinal nodes, and adrenal, frontal skull bone, and left pelvic bone metastases (T4N2M1c). She underwent an FDG-PET scan as part of her staging investigations in June 2017, revealing an area of intense heterogenous FDG-PET avidity in the sigmoid colon. This was suspicious for a metastatic deposit or a complication secondary to diverticular disease (Figure 1). However, a colonoscopy done 6 months prior had been normal. A CT scan was performed which demonstrated a focal area of thickening of the sigmoid colon (Figure 2); however, given the recent colonoscopy findings, the possibility of malignancy was deemed less likely in this situation.

The patient had minimal comorbidities and palliative systemic treatment, including radiation, was organised. She proceeded to carboplatin plus gemcitabine chemotherapy and completed 4 cycles in September 2017. She received palliative radiation to the right frontal bone and left pelvis metastatic deposits. She was then commenced on maintenance pemetrexed chemotherapy in October 2017.

In March 2018, she had a repeat colonoscopy, which revealed two polyps and evidence of diverticulosis in the sigmoid and descending colon. The polyps were removed, and histopathology revealed no evidence of malignancy.

In April 2018, she developed asymptomatic low-volume brain metastases in the left temporal, left occipital, and right posterior frontal lobes ranging from 3 mm to 16 mm in diameter. She underwent gamma knife treatment to these lesions and proceeded to Nivolumab immunotherapy in April 2018.

After 2 cycles of Nivolumab, our patient developed mild lower abdominal pain, which she complained of during her outpatient oncology visits. This had been diagnosed as diverticulitis by her general practitioner, who commenced antibiotic treatment.

**Figure 3:**
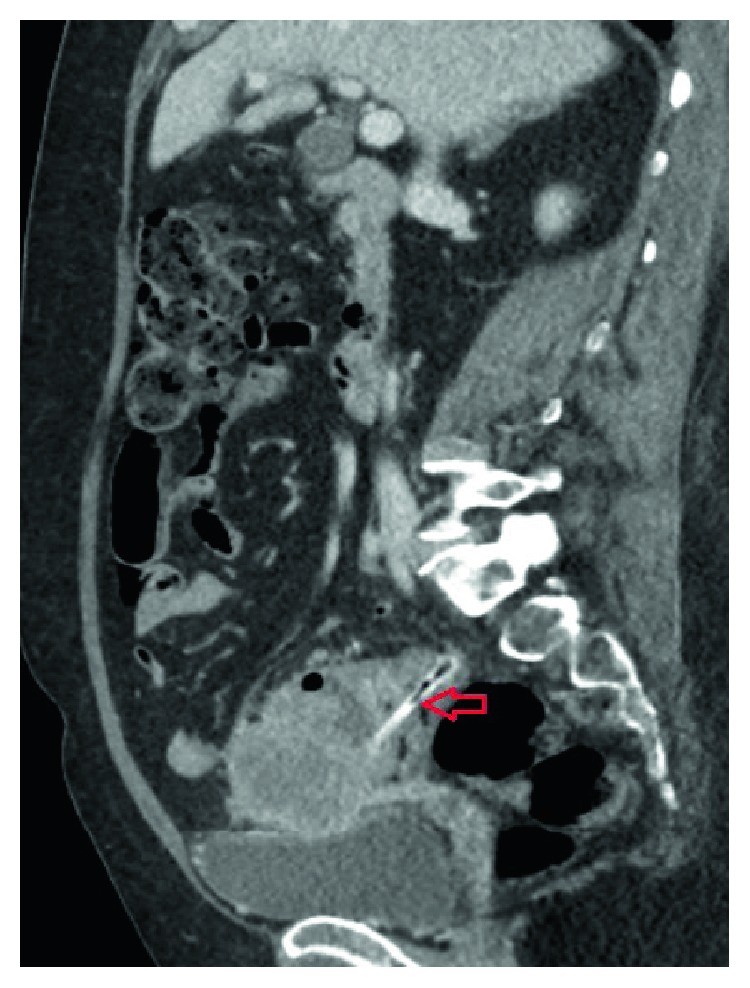
Sagittal CT highlighting a hyperdense tubular foreign object in the sigmoid colon (arrow), in addition to displaying a mass-like thickening of the sigmoid colon.

**Figure 4:**
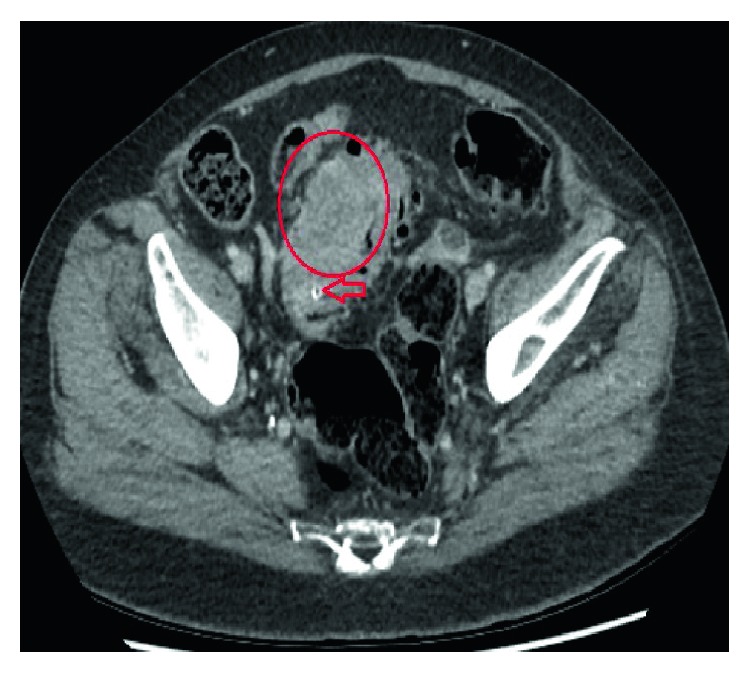
Axial CT scan. Arrow points out a cross-sectional image of the foreign body in question. Meanwhile, the area outlined represents the mass-like thickening of the sigmoid colon proximal to the foreign body.

A CT scan demonstrated circumferential thickening of the bowel wall in the sigmoid colon and a suspicious-looking intraluminal tubular structure distal to this, suspicious for a foreign body (Figures 3 and 4). The patient could not remember ingesting anything unusual or ingesting a bone. She, also, did not have any further colonic instrumentation after her colonoscopy. There was some thought that this may have been a clip from her colonoscopy, although the appearance of the foreign body was not consistent with this. Nivolumab was ceased and antibiotics were continued.

**Figure 5:**
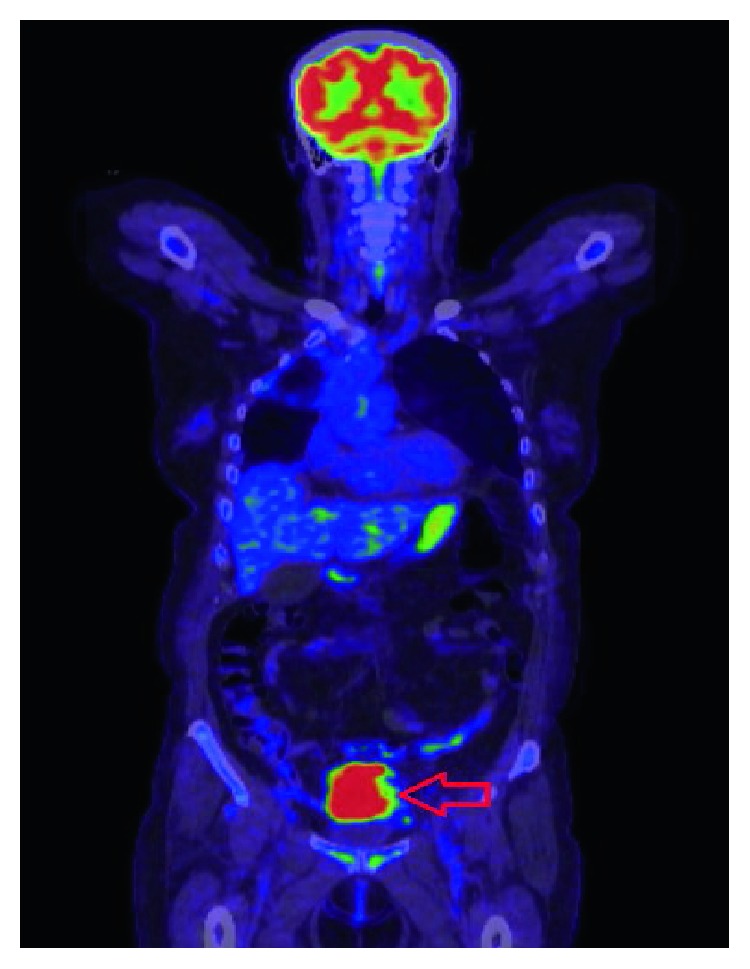
FDG-PET scan of our patient, following two weeks of serial radiological imaging, to monitor the foreign body. An intensely FDG-PET avid mass in the sigmoid colon was highlighted on imaging (arrow).

The patient continued to eat normally during this time and reported no changes in her bowel habits. She had no fevers and the only abnormality on her blood results was a raised C-reactive protein. The clinical decision was to follow this closely with serial imaging. Progress imaging 2 weeks later confirmed persistence of this foreign body. Consequently, our patient was admitted due to ongoing lower abdominal and suprapubic pain and for intravenous antibiotics. A repeat FDG-PET-CT scan was conducted, revealing an intensely FDG avid mass in the midsigmoid colon (Figure 5). The increase in size of the mass was concerning for a primary neoplasm or an extramural metastatic deposit from our patient's advanced lung cancer, given she had a colonoscopy which revealed no mucosal neoplasm.

**Figure 6:**
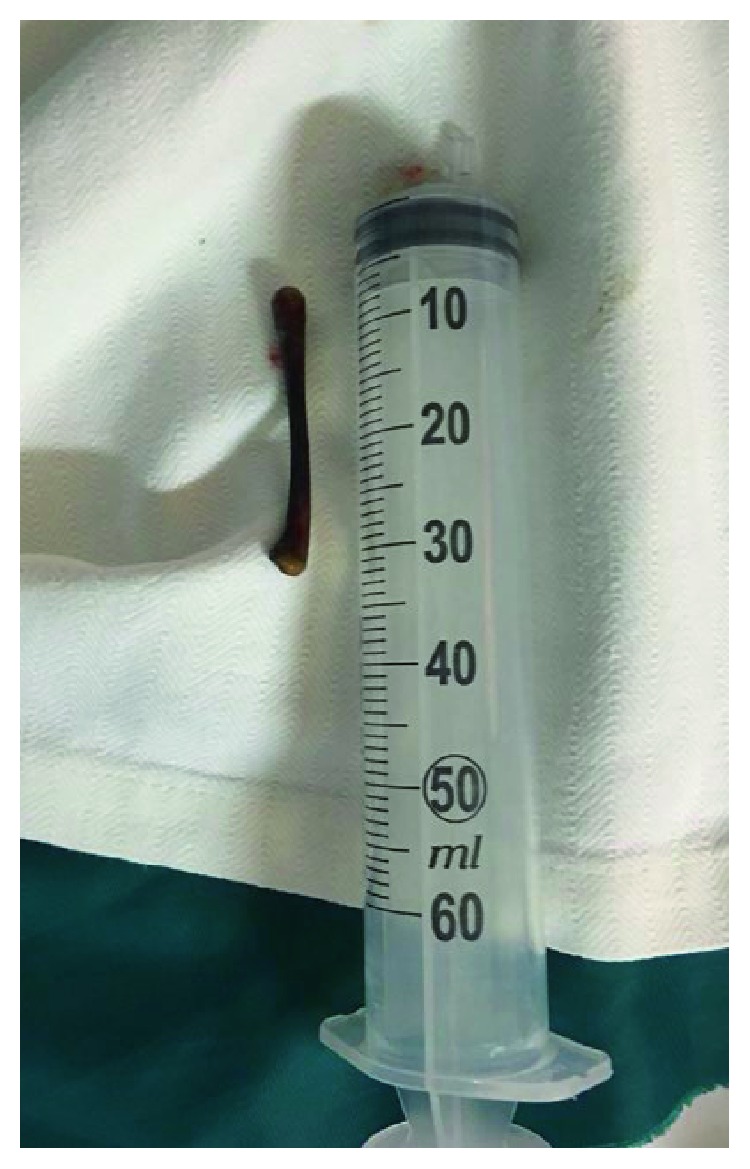
Image of chicken bone retrieved with flexible sigmoidoscopy. The bone measured 6 cm in length with no apparent sharp edges.

Despite these findings, it was still possible that this was secondary to an inflammatory rather than a neoplastic process. The patient was scheduled for a flexible sigmoidoscopy to evaluate the intracolonic foreign body. This revealed a chicken bone impacted in the sigmoid colon (Figure 6). The extent of the inflammation was such that the scope could not be passed 10 cm beyond the chicken bone. Nevertheless, the bone was easily removed with a snare. Imaging was conducted after 3 days to ensure there was no perforation or complication, as a result of procedure, given our patient's concomitant chemotherapy, following which she was discharged.

**Figure 7:**
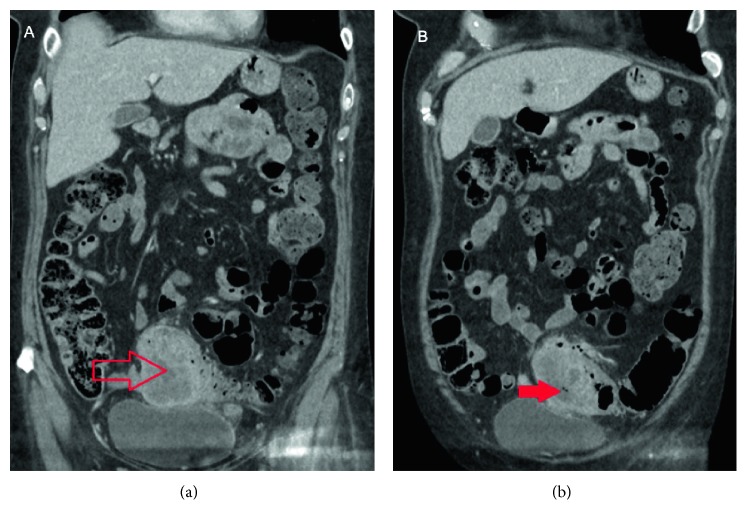
Coronal CT of the abdomen and pelvis highlighting an intramural sigmoid mass (a). Furthermore, the appearance of abscess transformation was noted, with gas locules evident (b).

The patient unfortunately represented the day after discharge with a hip fracture following a mechanical fall. She underwent a hip replacement and during her postoperative recovery developed more abdominal pain. A further CT scan raised concern that this mass had become an intramural abscess with images displaying some gas locules within it (Figure 7). She was managed with further intravenous antibiotics for 2 weeks. Progress imaging had revealed little change in the mass, and the antibiotics were ceased.

**Figure 8:**
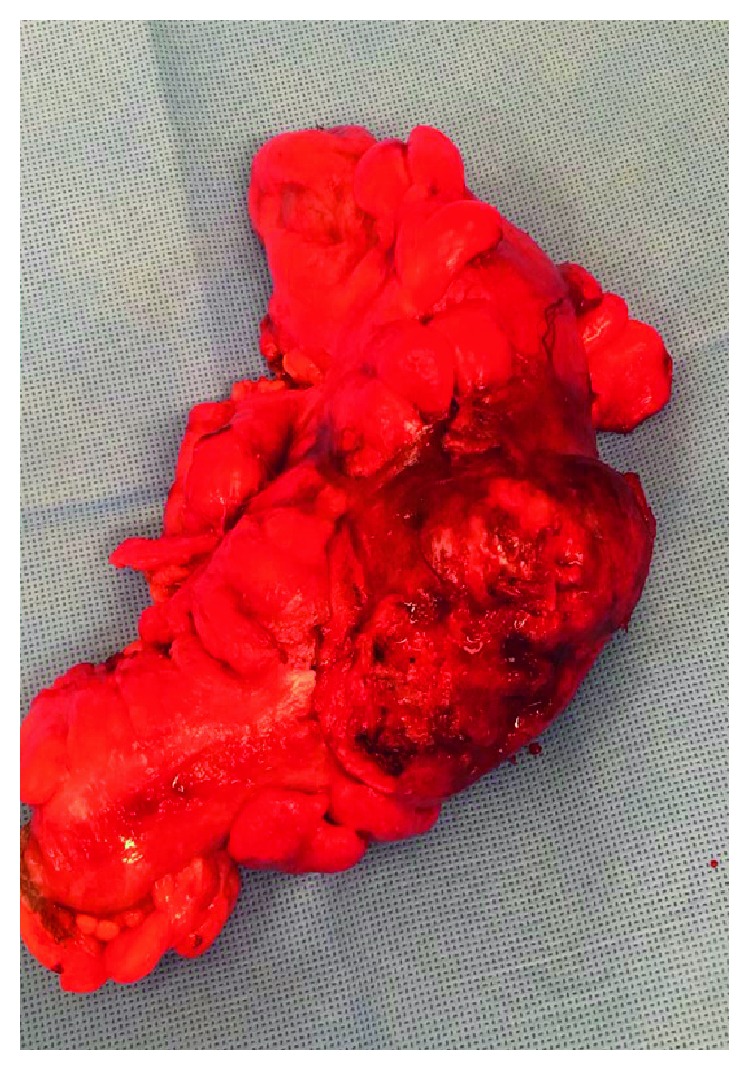
Image of resected sigmoid mass, following laparotomy. Histopathology confirmed the mass to be metastatic lung cancer.

**Figure 9:**
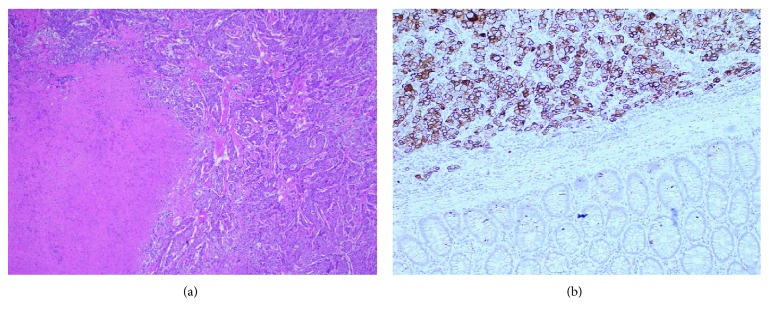
Photomicrograph of patient's resected sigmoid mass. (a) H&E staining displaying atypical tumour cells and areas of necrosis under 100x magnification. (b) Specimen under 100x magnification with CK7 staining outlining a diffuse distribution of tumour cells.

She was discharged and remained well the first week following her discharge. The following week, she developed worsening pain, fevers, and a subacute large bowel obstruction. She underwent an emergency laparotomy, at which time, she was found to have a large, fungating, and hard mass, which was densely adherent to the bladder. She underwent a resection of this sigmoid mass along with a contiguous segment of the bladder (Figure 8). The segment of the bladder was repaired, and an end colostomy was fashioned. Histopathology confirmed that this mass was a large deposit of metastatic lung cancer (Figure 9).

Unfortunately, during the course of her recovery, our patient had another fall and broke her other hip. She has since had this hip replaced and has recovered from her surgery and is managing her stoma. She underwent further rehabilitation and was discharged home. She remains on systemic treatment for metastatic lung cancer.

## 3. Discussion

Our case represents a rare and unusual presentation of an impacted chicken bone in the setting of a sigmoid mass. Thirty-six reports of complications as a result of chicken bones in the large bowel were identified in the English literature (Table 1). The sigmoid colon was implicated in 22 of these 36 case reports. This is not surprising as the rectosigmoid junction represents one of the narrowest regions in the gastrointestinal tract and hence represents the more likely area where complications from ingested foreign bodies may present [3].

In our review of the 36 reported cases of complications from chicken bone ingestion, nonspecific abdominal pain was a common presenting complaint throughout, in similar fashion to how our patient presented. Radiology formed a cornerstone in the workup of patients with ingested foreign bodies, with CT and X-ray of the abdomen standing out as the most common investigations organised. Ultimately, endoscopy served as the most common means of gaining a definitive diagnosis, while concomitantly managing the condition. Surgery, however, was necessary in cases where the chicken bone had led to serious complications.

In terms of these complications from ingested chicken bones, bowel perforation was noted to occur in 19 of the 36 case reports analysed. A history of gastrointestinal disease, such as diverticulosis and colonic malignancy, predisposes individuals to experiencing complications of ingested foreign bodies, especially that of perforation [1]. In our patient, the foreign body persisted in its location in the sigmoid colon just beyond the mass and did not cause a perforation despite her known diverticulosis.

A review of the literature revealed that patients with a history of alcoholism, dentures, or sensory neuropathy are most at risk of swallowing a foreign body [2]. Our patient did have dentures, which may very well have predisposed her to accidentally ingesting a chicken bone, which at the time she could not recall.

Our patient was on an immune checkpoint inhibitor for her lung cancer. Immune checkpoint inhibitors have known immune-related gastrointestinal toxicities such as diarrhoea and colitis. Rare cases of bowel perforation requiring colostomy have been reported in the literature [38]. The development of an intramural sigmoid abscess in our patient following the chicken bone removal could be solely attributed to the chicken bone impaction and subsequent removal. It is possible that the impacted chicken bone may have affected or breached the luminal integrity of the bowel focally leading to abscess formation on its removal. Equally, hypothetically, tumour response in the metastatic lung cancer deposit in the sigmoid colon could have contributed to some degree to a breach in the colonic integrity and the formation of the intramural abscess. In our patient, both factors may have played a role in the abscess formation and ensuing colonic obstruction that necessitated surgery.

To our knowledge, this is the first case of chicken bone impaction in the setting of metastatic lung cancer to the sigmoid colon. Some of the difficulties, even with modern imaging and FDG-PET, in differentiating inflammatory from neoplastic processes in the bowel are described. This case highlights that while colonoscopy is useful in visualising the bowel from within and is crucial in diagnosing malignancies arising from the bowel mucosa, its ability in diagnosing extramural malignancies arising beyond or external to the mucosa of the bowel as in the case of metastatic extramural disease can be limited.

## 4. Conclusion

Foreign bodies mostly present with nonspecific abdominal pain, and while the majority are managed surgically, they can sometimes be retrieved endoscopically. In the large bowel, the sigmoid colon is the most common site of complications arising from ingested chicken bones. The literature review identified that perforation of the bowel tends to occur in the setting of diverticular disease and malignancy. Our case reflects the diagnostic complexity in a patient with an ingested foreign body in the setting of metastatic disease, despite modern radiological investigative modalities and endoscopy. This report highlights the value of keeping ingested foreign bodies in mind when formulating differential diagnoses for nonspecific abdominal pain. At the same time, it identifies a key area in oncological practice, where rigorous follow-up is essential to the screening for metastasis of primary malignancies to distant organ sites.

## Figures and Tables

**Table 1:** Case reports of ingested chicken bones in the large bowel derived from the English literature [3–37].

Author and country	Patient	Presentation	Investigations	Diagnosis	Management
Glasson et al. [3]; Wagga Wagga, Australia	70-year-old, male	(i) Abdominal pain (ii) Weight loss (iii) Altered bowel habits	(i) Full blood count (ii) CT abdomen (iii) Abdominal X-ray (iv) Laparotomy	Perforated sigmoid diverticulum with fibrous adhesions to the ileocaecal junction	Subtotal colectomy with ileorectal anastomosis

Werner and Gallegos-Orozco [4]; Arizona, USA	65-year-old, female	(i) Fatigue (ii) Nausea (iii) Pyrexia	(i) Abdominal examination (ii) Full blood count (iii) CT	Sigmoid perforation with hepatic abscesses	Colonoscopy and antibiotics

McGregor et al. [5]; Kansas, USA	86-year-old, male	(i) Left-sided abdominal pain (ii) Vomiting (iii) Anorexia	(i) Abdominal X-ray (ii) Colonoscopy	(i) Sigmoid perforation with peritonitis and adhesions (ii) Underlying adenocarcinoma	Sigmoid resection with end colostomy and Hartmann's pouch

Girelli and Colombo [6]; Arsizio, Italy	70-year-old, male	(i) Severe rectal bleeding	(i) Endoscopy (ii) Colonoscopy	Bone impacted in hepatic flexure	Removal with polypectomy snare

Coyte et al. [7]; Glasgow, UK	76-year-old, male	(i) Abdominal pain (ii) Vomiting (iii) Pyrexia	(i) Erect chest X-ray (ii) CT	Small and large bowel perforation	Resection of midjejunum and sigmoid

Terrace et al. [8]; Edinburgh, UK	85-year-old, male	(i) Left lower quadrant pain (ii) Diarrhoea	(i) Erect chest X-ray (ii) CT abdomen and pelvis	Sigmoid perforation with distal adenocarcinoma	Anterior resection with colorectal anastomosis

Mesina et al. [9]; Craiova, Romania	52-year-old, female	(i) Left perianal pain with swelling (ii) Pyrexia	(i) Physical examination	Ischiorectal abscess	Tear-drop incision

Cardoso et al. [10]; Setubal, Portugal	80-year-old, male	(i) Vomiting (ii) Diarrhoea (iii) Pyrexia	(i) Full blood count (ii) Abdominal ultrasound (iii) CT	Hepatic abscess, bone located in the ascending colon	Colonoscopy

Park et al. [11]; Seoul, South Korea	68-year-old, female	(i) Anal pain and bleeding (ii) Constipation	(i) Digital rectal examination (ii) Abdominal X-ray (iii) CT abdomen and pelvis	Stercoral ulcer of the rectum	Flexible sigmoidoscopy and sucralfate enema postoperatively

Akhtar et al. [12]; Belfast, UK	46-year-old, male	(i) Abdominal pain (ii) Vomiting	(i) Full blood count (ii) Erect chest X-ray	Sigmoid perforation	Laparotomy with repair of perforation

Glasson et al. [3]; Wagga Wagga, Australia	70-year-old, male	(i) Abdominal pain (ii) Weight loss (iii) Altered bowel habits	(i) Full blood count (ii) CT abdomen (iii) Abdominal X-ray (iv) Laparotomy	Perforated sigmoid diverticulum with fibrous adhesions to the ileocaecal junction	Subtotal colectomy with ileorectal anastomosis

Vardaki et al. [13]; Athens, Greece	69-year-old, male	(i) Abdominal pain	(i) Full blood count (ii) CT abdomen	Sigmoid perforation with underlying carcinoma	Open surgery

Rasheed et al. [14]; Massachusetts, USA	59-year-old, male	(i) Left lower quadrant pain	(i) CT abdomen	Sigmoid perforation	Surgical management

Kornprat et al. [15]; Graz, Austria	82-year-old, female	(i) Sepsis (ii) Severe abdominal pain	(i) Full blood count (ii) CT abdomen and pelvis	Perforated sigmoid diverticulum; phlegmonous inflammation of the abdominal wall	Emergency Hartmann's procedure with necrectomy of the abdominal wall

Clements et al. [16]; Virginia, USA	66-year-old, male	(i) Sepsis (ii) Anuria	(i) Urine/blood cultures (ii) Renal ultrasound (iii) CT KUB (iv) Colonoscopy	Colovesical fistula with submucosal/intramural haemorrhage in the sigmoid colon	Low anterior resection with primary anastomosis and bladder repair

Tay et al. [17]; Singapore	73-year-old, male	(i) Irreducible left inguinal hernia (ii) Abdominal pain	(i) CT abdomen (ii) Exploratory laparotomy	Perforated sigmoid colon	Sigmoid colectomy

Joglekar et al. [18]; Great Yarmouth, UK	47-year-old, male	(i) Abdominal pain (ii) Diarrhoea	(i) Full blood count (ii) Urine dipstick (iii) Laparotomy	Perforated sigmoid colon	Repair of perforation

Bleich [19]; Connecticut, USA	54-year-old, female	(i) Left lower quadrant pain (ii) Pyrexia	(i) Full blood count (ii) CT abdomen (IV and oral contrast)	Impacted chicken bone in sigmoid diverticulum	Flexible sigmoidoscopy and oral antibiotics

Brucculeri et al. [20]; Monserrato, Italy	75-year-old, female	Lower abdominal pain	(i) CT abdomen (with and without contrast) (ii) Flexible sigmoidoscopy	Impacted chicken bone across the diameter of the colon wall	Laser source contact and removal of divided bone with forceps

Domínguez-Jiménez and Jaén-Reyes [21]; Andujar, Spain	79-year-old, female	Asymptomatic, presenting for programmed colonoscopy	(i) Colonoscopy (ii) Subsequent CT abdomen	Perforated sigmoid diverticulum with thickening of the right pelvic fascia	Conservative management—patient expelled bone in faeces after 2 months

Khan et al. [22]; Craigavon, Northern Ireland	56-year-old, male	(i) Painful haematuria (ii) Polyuria (iii) Pneumaturia	(i) Urine culture (ii) IV urogram (iii) CT scan (iv) Cystoscopy	Colovesical fistula, secondary to perforated colon wall	Surgical exploration with resection of perforated bowel

Lubel and Wiley [23]; Woodville South, Australia	54-year-old, female	(i) Persistent lower abdominal pain (ii) Rectal mucous	(i) Colonoscopy	Chicken bone impacted in inflamed diverticula	Laparotomy with sigmoid resection

Mapelli et al. [24]; Louisiana, USA	72-year-old, female	(i) Left lower quadrant pain (ii) Anorexia	(i) Abdominal ultrasound (ii) Colonoscopy	Perforation of the sigmoid colon	Resection of the sigmoid colon

Milivojevic et al. [25]; Belgrade, Serbia	75-year-old, female	(i) Nausea (ii) Left lower quadrant pain (iii) Fever	(i) Abdominal X-ray (ii) Colonoscopy	Impacted chicken bone in the sigmoid colon	Colonoscopy

Owen et al. [26]; London, UK	65-year-old, male	(i) Severe lower abdominal pain (ii) Dehydration (iii) Pyrexia	(i) Erect chest X-ray (ii) CT abdomen (iii) Laparoscopy	Sigmoid perforation	Colonoscopy with insertion of abdominal drain

Rabb et al. [27]; South Yorkshire, UK	69-year-old, male	(i) Asymptomatic (ii) Bowel cancer screening (positive for faecal occult blood)	(i) Colonoscopy	Impaction of chicken bone in bowel diverticulum	Laparoscopic sigmoid colectomy

Rex and Bilotta [28]; Indiana, USA	73-year-old, male	(i) Lower abdominal pain	(i) Colonoscopy	Impacted chicken bone across two diverticula	Colonoscopy

Rex and Bilotta [28]; Indiana, USA	81-year-old, female	(i) Lower abdominal pain (ii) Positive faecal occult blood	(i) Barium enema (ii) Colonoscopy	Impacted chicken bone in sigmoid diverticula	Colonoscopy

Tarnasky et al. [29]; North Carolina, USA	80-year-old, female	(i) Chronic diarrhoea (ii) Positive faecal occult blood	(i) Abdominal examination (ii) Colonoscopy	Perforated sigmoid colon, due to impacted chicken bone	Colonoscopy

Chen et al. [30]; Sydney, Australia	84-year-old, female	(i) Lower abdominal pain	(i) Colonoscopy (ii) CT abdomen	Impacted chicken bone across the diameter of the colon lumen	Nd:YAG laser/colonoscopy

Ross et al. [31]; Glasgow, UK	87-year-old, female	(i) Severe bleeding per anus	(i) CT abdomen with angiogram	Impacted chicken bone in sigmoid diverticulum with arterial bleeding	Flexible sigmoidoscopy

Elmoghrabi et al. [32]; Michigan, USA	70-year-old, female	(i) Lower abdominal, pelvic, and rectal pain	(i) Abdominal X-ray (ii) CT abdomen/pelvis	Large rectal stricture secondary to impacted chicken bone	Resection of the rectum and distal sigmoid colon

Davies [33]; Cardiff, UK	31-year-old, male	(i) Severe rectal pain	(i) Abdominal X-ray	Perforated rectum immediately proximal to anal margin	Digital removal followed by proctosigmoidoscopy

Jeen et al. [34]; Seoul, South Korea	73-year-old, female	(i) Abdominal cramping (ii) Diarrhoea	(i) Colonoscopy	Chicken bone impaction in the sigmoid colon across the lumen diameter	Balloon dilatation and extraction

Osler et al. [35]; New York, USA	78-year-old, female	(i) Abdominal pain (ii) Nausea (iii) Vomiting	(i) CT abdomen (ii) Exploratory laparotomy	Sigmoid perforation distal to colonic carcinoma	Hartmann's resection with end sigmoid colostomy

Moreira et al. [36]; Pennsylvania, USA	31-year-old, male	(i) Pain around the anal canal and scrotum (ii) Pyrexia	(i) Open surgery	Necrotising fasciitis with perianal and scrotal abscesses	Debridement and antibiotic therapy

Muñoz et al. [37]; Baracaldo, Spain	67-year-old, male	(i) Left lower quadrant abdominal pain (ii) Tenesmus	(i) Abdominal X-ray (ii) Barium enema	Impacted chicken bone in the sigmoid colon	Colonoscopy
